# Curcumin as a Green Antibiotic Substitute: Mechanisms and Applications in Poultry Production and Health Promotion

**DOI:** 10.3390/ani16081242

**Published:** 2026-04-17

**Authors:** Xiaopeng Tang, Baoshan Zhang, Jiayuan Yang, Youyuan Xie, Kangning Xiong

**Affiliations:** State Engineering Technology Institute for Karst Desertfication Control, School of Karst Science, Guizhou Normal University, Guiyang 550025, China; 13688589056@163.com (B.Z.); 15202882842@163.com (J.Y.); xxsc9920@163.com (Y.X.); xiongkangning2021@126.com (K.X.)

**Keywords:** antibiotic alternative, anti-inflammatory, antioxidant, biological functions, curcumin, poultry production

## Abstract

Against the backdrop of the full implementation of “antibiotic ban” and “zinc restriction” policies in livestock and poultry breeding, and the growing consumer demand for safe livestock and poultry products, the development of natural and efficient green feed additives has become crucial for the sustainable development of the animal husbandry industry. Curcumin, a natural polyphenolic compound extracted from the rhizome of *Curcuma longa* L., has attracted extensive attention in poultry production due to its various biological activities and safety. This paper thoroughly reviews the chemical structure and physicochemical properties of curcumin, and elaborates on its core molecular mechanisms of action, which mainly involve the regulation of nuclear factor erythroid 2-related factor 2 (Nrf2)/antioxidant response element (ARE), nuclear factor-κB (NF-κB), peroxisome proliferator-activated receptor γ (PPAR-γ), and mitogen-activated protein kinase (MAPK) pathways to exert antioxidant, anti-inflammatory, antibacterial, immunomodulatory and lipid metabolism regulatory effects. It further clarifies the practical application value of curcumin in major poultry species including broilers, laying hens, ducks and quails, showing that curcumin can significantly improve poultry production performance, optimize meat and egg quality, protect intestinal health, and enhance the ability of poultry to resist stress and diseases. Meanwhile, the review notes curcumin’s current application limitations (low bioavailability, poor stability, unclear standardized dosage, and high industrialization cost) and proposes targeted future research directions to address these issues. In conclusion, curcumin is a promising green feed additive alternative to antibiotics, and its large-scale and standardized application in poultry production will effectively promote the green, healthy and sustainable development of the poultry industry.

## Simple Summary

With the implementation of antibiotic and zinc restriction policies in poultry farming, curcumin, a natural polyphenol from turmeric, has become a promising antibiotic substitute for its diverse biological activities. This paper explores how curcumin acts on key signaling pathways to exert antioxidant, anti-inflammatory and antibacterial effects, and details its positive role in improving production performance, product quality and disease resistance of poultry. It also points out the practical limitations of curcumin in applications such as low bioavailability and poor stability, and puts forward targeted research and improvement directions for its large-scale use in the poultry industry.

## 1. Introduction

Curcumin is a natural polyphenolic compound extracted from the rhizome of *Curcuma longa*, a plant in the Zingiberaceae family [1]. As a plant active ingredient with a long history of application, curcumin has been widely used in traditional Chinese medicine for anti-inflammatory, antioxidant, wound healing and other therapeutic purposes [2]. With the development of analytical and molecular biological techniques, the diverse biological functions of curcumin have been clarified, extending its applications to food, cosmetics and animal production [2,3,4].

During animal husbandry development, the issues of bacterial resistance and drug residues resulting from excessive antibiotic use have become increasingly severe, making the search for safe and effective antibiotic alternatives an urgent industry need [5]. To address these challenges, natural plant-derived feed additives with multiple biological functions and high biosafety have become the focus of current research. Among them, curcumin shows unique advantages and broad application potential. Curcumin, with its broad-spectrum biological activities, good safety, environmental friendliness, and significant antioxidant, anti-inflammatory, antibacterial, immunomodulatory, and growth-promoting functions, is considered a promising candidate for antibiotic replacement that can effectively improve animal health and production performance [3,6]. Especially under the current policy background of “zinc restriction” and “antibiotic ban”, particularly regarding their use in agriculture and animal feed, the application prospect of this natural plant extract is broader.

However, curcumin has poor water solubility, low bioavailability, and is sensitive to light, heat, and alkaline environments, making it prone to degradation during feed processing and storage [7,8]. These disadvantages have significantly restricted the practical application of curcumin in feed production. To overcome these problems, researchers have developed new preparation technologies such as nanoparticles, metal complexes and cyclodextrin inclusion complexes [8,9,10]. These strategies can enhance water solubility, reduce degradation caused by light, heat and alkaline conditions, promote intestinal absorption, and extend its effective duration in vivo, thus significantly improving the stability and oral bioavailability of curcumin. This paper thoroughly reviews the chemical structure characteristics and biological functions of curcumin and its research progress in poultry production, focusing on its mechanism of action, application limitations and future directions to provide a theoretical basis and practical guidance for the scientific application of curcumin in animal production.

## 2. Chemical Structure and Physicochemical Properties of Curcumin

**Figure 1:**
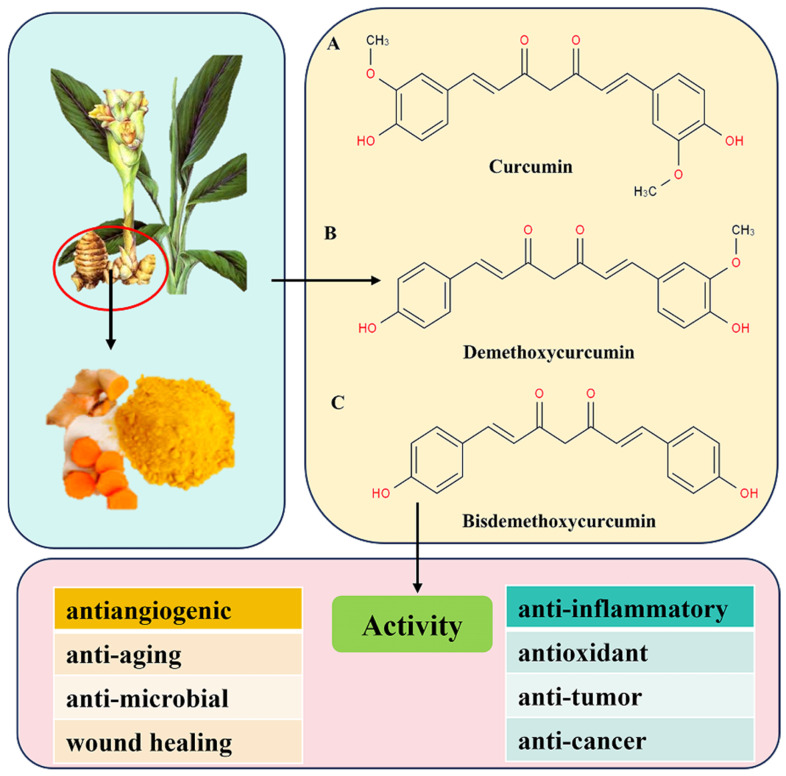
Chemical structures and biological activities of main curcuminoids. The structures include curcumin, demethoxycurcumin, and bisdemethoxycurcumin. The phenolic hydroxyl (-OH), methoxy (-OCH3) on the benzene rings, and β-diketone group in the heptane chain are core functional groups responsible for their biological activities, including anti-inflammatory, anti-angiogenic, antioxidant, anti-aging, anti-tumor, anti-microbial, anti-cancer and wound healing effects [6,7,11].

Curcumin is a hydrophobic polyphenolic compound extracted from the rhizome of *Curcuma longa*, with the chemical name 1,7-bis(4-hydroxy-3-methoxyphenyl)-1,6-heptadiene-3,5-dione (molecular formula C_21_H_20_O_6_, molecular weight 368.37 g/mol), belonging to the diarylheptanoid class of compounds [6]. Its core structure consists of two aromatic rings (benzene rings) connected by a heptane chain, with hydroxyl (-OH), methoxy (-OCH_3_) and β-diketone groups. Among them, the β-diketone group is an important active center, which enables it to undergo tautomerism, affecting its chemical properties and biological activities [11]. The keto-enol tautomerism of curcumin depends on the acidity of the solution. It mainly exists in the keto form in acidic and neutral media and the enol form in alkaline media [7]. Natural *Curcuma longa* extracts usually contain three main curcuminoids: curcumin, demethoxycurcumin, and bisdemethoxycurcumin (Figure 1).

Curcumin is an orange-yellow crystalline powder with low water solubility but good solubility in organic solvents such as ethanol, ether and acetone [7,12]. It possesses poor chemical stability and is sensitive to light and heat, leading to gradual degradation under long-term exposure; meanwhile, it is prone to isomerization under alkaline conditions [13,14,15]. Furthermore, curcumin is hardly absorbed by intestinal epithelial cells, rapidly metabolized in the liver, and quickly eliminated from the systemic circulation [16]. Collectively, these physicochemical and metabolic characteristics result in its low bioavailability, which has become a major limiting factor for its application in animal production.

## 3. Biological Functions of Curcumin

### 3.1. Mechanism of Antioxidant Action of Curcumin

**Figure 2:**
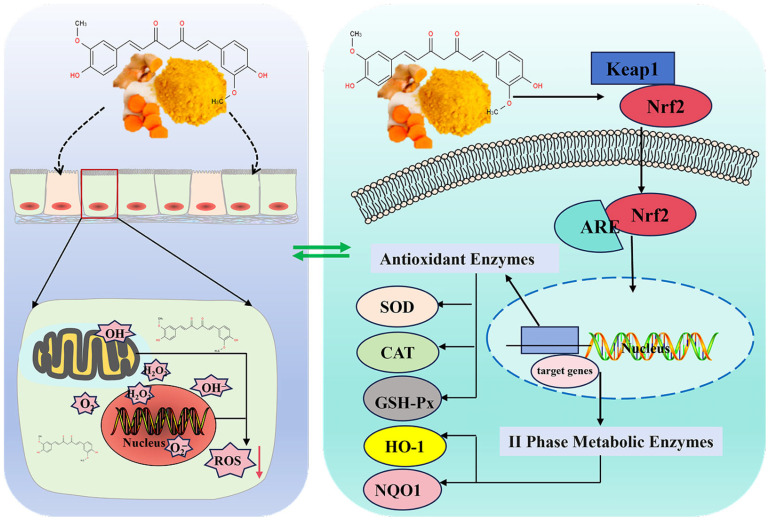
Schematic diagram of curcumin regulating the Nrf2/ARE antioxidant signaling pathway. Curcumin enters the cell and interacts with Keap1, dissociating Nrf2 from the Keap1-Nrf2 complex. The free Nrf2 translocates into the nucleus and binds to the ARE in the promoter region of target genes. This binding activates the transcription and expression of downstream antioxidant enzyme genes (SOD, CAT, and GSH-Px) and phase II metabolic enzyme genes (HO-1 and NQO1), thereby enhancing the cell’s ability to scavenge reactive oxygen species (ROS) and reducing oxidative damage [6,17,18,19].

Curcumin, a highly effective natural antioxidant, functions through multiple molecular targets and signaling pathways, primarily via two major mechanisms (Figure 2). Firstly, it directly scavenges reactive oxygen species (ROS). Excessive ROS accumulation often leads to mitochondrial dysfunction and further oxidative stress injury [6]. The phenolic hydroxyl and β-diketone structures within its molecular framework endow curcumin with remarkable electron-donating capacity, enabling it to react directly with free radicals and mitigate oxidative damage [17]. Meanwhile, curcumin can maintain mitochondrial structural integrity, reduce mitochondrial ROS production, and regulate mitophagy to remove damaged mitochondria, thereby further enhancing its antioxidant and cytoprotective effects [18].

Second, curcumin activates the endogenous antioxidant system by regulating the nuclear factor erythroid 2-related factor 2 (Nrf2)/antioxidant response element (ARE) signaling pathway [6,18]. It inhibits the Kelch-like ECH-associated protein 1 (Keap1)-Nrf2 interaction, promotes Nrf2 nuclear translocation, and enhances ARE activation, which subsequently upregulates the expression of phase II metabolic enzyme genes, including heme oxygenase (HO-1) and NADP(H):quinone oxidoreductase (NQO1), as well as antioxidant enzyme genes such as superoxide dismutase (SOD), glutathione peroxidase (GSH-Px) and catalase (CAT), thereby enhancing the cellular antioxidant defense capacity [3,18,19].

### 3.2. Anti-Inflammatory Effects and Molecular Mechanisms of Curcumin

#### 3.2.1. Curcumin Inhibits the Nuclear Factor-κB (NF-κB) Signaling Pathway

**Figure 3:**
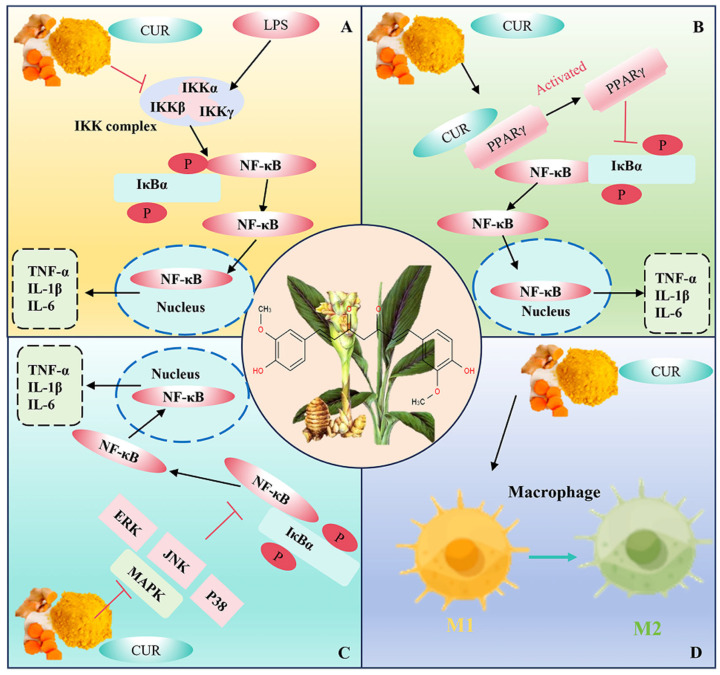
Multi-pathway mechanisms of curcumin’s anti-inflammatory effects in poultry. Curcumin exerts anti-inflammatory effects through four main pathways: (**A**) Curcumin inhibits the IKK complex, preventing the phosphorylation (P) and degradation of IκBα. This keeps NF-κB bound to IκBα in the cytoplasm, blocking its nuclear translocation and subsequent transcription of pro-inflammatory cytokines (TNF-α, IL-1β, IL-6) [24,25,26]. (**B**) Curcumin activates PPARγ, which then inhibits the nuclear translocation of NF-κB, thereby reducing the expression of pro-inflammatory factors [28,29,30,31]. (**C**) Curcumin interferes with the activation of MAPK pathway components (JNK, ERK, p38), inhibiting their phosphorylation and subsequent downstream signaling that promotes inflammation [35,36,37,38]. (**D**) Curcumin regulates macrophage polarization, shifting the balance from pro-inflammatory M1 macrophages to anti-inflammatory M2 macrophages, thereby reducing the production of pro-inflammatory cytokines and enhancing anti-inflammatory effects [41,42].

NF-κB, a key transcription factor, plays a central role in mediating the inflammatory response. Extrinsic stimuli such as lipopolysaccharide (LPS) and mycotoxins can activate the NF-κB pathway and promote the expression of pro-inflammatory genes [20,21]. Toll-like receptor 4 (TLR4) is a critical upstream regulator of the NF-κB pathway, serving as the primary sensor for pathogen-associated molecular patterns (PAMPs) and damage-associated molecular patterns (DAMPs) released by damaged mitochondria [5,6]. Curcumin exerts its anti-inflammatory effects by directly inhibiting TLR4 activation, thereby blocking the TLR4/NF-κB inflammatory cascade at the source [22,23]. Specifically, curcumin inhibits the activity of IκB kinase (IKK), preventing the phosphorylation and degradation of nuclear factor of kappa light polypeptide gene enhancer in B-cells inhibitor α (IκBα). As a result, NF-κB remains bound to IκBα and is retained in the cytoplasm, which inhibits its translocation to the nucleus and the subsequent activation of downstream inflammatory genes. This mechanism effectively reduces the production of pro-inflammatory cytokines such as interleukin (IL)-1β, IL-6 and tumor necrosis factor-α (TNF-α) (Figure 3A) [24,25,26]. This inhibition not only suppresses pro-inflammatory cytokine expression but also interrupts the positive feedback loop between inflammation, ROS production, and mitochondrial dysfunction, synergistically enhancing curcumin’s cytoprotective effects.

The antioxidant Nrf2 pathway and the pro-inflammatory NF-κB pathway exhibit extensive crosstalk, which is tightly linked to mitochondrial function and mitophagy. Curcumin acts as a dual regulator of this network: it activates Nrf2 to upregulate antioxidant enzymes, reducing ROS levels and mitochondrial damage [3,18,19]; simultaneously, it inhibits TLR4/NF-κB signaling, suppressing inflammatory responses [22,23,24,25,26]. Additionally, curcumin promotes mitophagy to clear damaged mitochondria, eliminating the source of excess ROS and pro-inflammatory DAMPs [17,18,23], which interrupts the positive feedback loop between inflammation, oxidative stress, and mitochondrial damage.

#### 3.2.2. Curcumin Activates Peroxisome Proliferator-Activated Receptor γ (PPAR-γ)

PPAR-γ is a nuclear receptor that plays an important role in regulating inflammatory responses and metabolic processes [27]. As a natural PPARγ agonist, curcumin can effectively activate this receptor, thereby exerting anti-inflammatory, metabolic regulatory, and tissue-protective effects [28]. Notably, PPAR-γ signaling is closely intertwined with the NF-κB pathway (the core inflammatory signaling pathway previously discussed), and their interaction is a key link in curcumin’s anti-inflammatory mechanism [28,29,30,31]. Specifically, activated PPAR-γ can directly bind to the NF-κB subunits (such as p65), inhibiting its nuclear translocation and subsequent binding to the promoter regions of pro-inflammatory genes (such as IL-1β, IL-6, and TNF-α), thereby suppressing the transcription and expression of these genes [29] (Figure 3B). For instance, in a rat model of Alzheimer’s disease (AD), curcumin directly binds to and activates PPARγ, which suppresses the NF-κB signaling pathway, reduces β-amyloid-induced neuroinflammation, and improves neuronal function and memory deficits in AD [28]. In an asthma model, curcumin alleviates airway inflammation and mucus secretion via the PPARγ-dependent NF-κB signaling pathway [30]. Additionally, curcumin inhibits cigarette smoke-induced inflammation by regulating the PPARγ-NF-κB signaling pathway [31].

#### 3.2.3. Curcumin Regulates the Mitogen-Activated Protein Kinase (MAPK) Pathway

The MAPK pathway is a core inflammatory signal transduction cascade that regulates multiple physiological processes, including cell growth, differentiation, apoptosis, and inflammatory responses [32]. This pathway consists of three primary branches, c-Jun N-terminal kinase (JNK), extracellular regulated protein kinases (ERK), and p38, all of which play critical roles in mediating pro-inflammatory gene expression [33]. Notably, the MAPK pathway exhibits extensive crosstalk with the NF-κB signaling pathway. Stress-activated MAPKs such as p38 and JNK, often via shared upstream activated mitogen-activated protein kinase kinase kinases (MAP3Ks) and adaptor proteins, such as transforming growth factor-activated kinase 1 (TAK1), contribute to the activation of the IκB kinase (IKK) complex, thereby promoting IκBα degradation and NF-κB nuclear translocation (e.g., p38 and JNK), forming a synergistic inflammatory amplification loop [34].

Curcumin exerts potent anti-inflammatory effects by targeting and inhibiting the activation of key MAPKs, including JNK, ERK, and p38 [35,36,37] (Figure 3C). This inhibition not only blocks MAPK-mediated inflammatory signaling but also disrupts the synergistic activation of NF-κB, thereby comprehensively suppressing the production of pro-inflammatory cytokines such as IL-1β, IL-6, and TNF-α [35,36,37]. For instance, in vascular smooth muscle cells, curcumin attenuates LPS-induced inflammation via NF-κB and JNK inhibition, with decreased p-JNK, p-c-Jun, p-p65, and p-IκBα and reduced inflammatory cytokines [38].

#### 3.2.4. Curcumin Regulates Macrophage Polarization

Macrophages are highly plastic immune cells that polarize into two distinct functional phenotypes in response to microenvironmental signals: the classically activated M1 (pro-inflammatory) phenotype, which secretes high levels of pro-inflammatory cytokines (e.g., IL-1β, IL-6, TNF-α) and chemokines to amplify inflammatory responses, and the alternatively activated M2 (anti-inflammatory, reparative) phenotype, which produces anti-inflammatory mediators and growth factors to promote tissue repair and immune homeostasis [39,40]. The balance between M1/M2 polarization is a critical determinant of inflammatory progression and disease outcome [40].

Curcumin exerts potent anti-inflammatory and tissue-protective effects by reprogramming macrophage polarization, primarily by suppressing M1 pro-inflammatory activation and promoting the switch toward the M2 anti-inflammatory phenotype [41,42] (Figure 3D). For instance, during malaria infection, curcumin decreases parasitemia and improves survival in infected mice by inhibiting M1 macrophage activation, thereby decreasing the production of pro-inflammatory cytokines that mediate immunopathology [41]. Additionally, in a mouse model of myocardial infarction, curcumin mitigates late ventricular remodeling by suppressing the inflammatory response in the early stages of the disease [42].

### 3.3. Antibacterial Effects of Curcumin

As a polyphenolic active substance, curcumin exhibits broad-spectrum antibacterial activity against various bacterial pathogens, including methicillin-resistant *Staphylococcus aureus*, *Escherichia coli*, *Pseudomonas aeruginosa*, *Enterococcus faecalis*, and *Porphyromonas gingivalis* [43,44,45]. Its antibacterial mechanisms involve damaging the integrity of bacterial cell walls and membranes, inhibiting the expression of virulence factors, biofilm formation, and bacterial adhesion to host receptors via the bacterial quorum sensing regulatory system. Additionally, curcumin acts as a photosensitizer, inducing phototoxicity under blue light irradiation. Its photolysis products, such as benzaldehyde and cinnamaldehyde, can disrupt bacterial gene expression, damage cell membranes, and inhibit dehydrogenase activity and ion gradient balance [46,47].

The biological functions of curcumin are closely linked to its chemical structure and are significantly influenced by external factors, including preparation form and concentration [48]. These multi-target and multi-pathway biological effects provide a robust theoretical foundation for its application in animal production.

## 4. Research Progress on the Application of Curcumin in Poultry Production

In the context of searching for natural feed additives to replace antibiotics, the application of curcumin in poultry production has emerged as a significant area of research. Numerous studies have demonstrated that curcumin possesses considerable potential for enhancing growth performance, meat quality, antioxidant capacity, immune function, and intestinal health in poultry [49,50,51].

### 4.1. Application Research in Broiler Production

As a natural feed additive, curcumin exerts positive effects on the production performance and intestinal health of broilers [49,51,52]. Studies have shown that the appropriate addition of curcumin can significantly improve the growth performance and feed utilization efficiency of broilers, enhance the intestinal morphological structure (reduce crypt depth (CD) and increase villus height (VH)), and promote nutrient absorption by regulating the intestinal microbial community (specifically, by promoting the proliferation of beneficial bacteria and inhibiting the growth of harmful bacteria), as well as enhancing intestinal barrier function and anti-inflammatory capacity [49,52,53,54,55,56,57,58]. In terms of meat quality, curcumin supplementation has been shown to improve carcass yield, improve meat color (with increased a* and b* values), enhance water-holding capacity, tenderness and juiciness, reduce cooking loss, fat content and lipid peroxide (MDA) levels, and ultimately boost the antioxidant capacity and oxidative stability of meat products [54,56,58,59,60]. Furthermore, curcumin can mitigate muscle fat deposition, optimize the fatty acid profile, and improve protein content and sensory quality by downregulating the expression of key genes associated with lipogenesis, including PPARγ and fatty acid synthase (FASN) [56,59,60,61].

Under stress or disease conditions such as heat stress [55,59,62,63,64,65], oxidative stress [66,67,68], high-density feeding [49], coccidiosis infection [57,69], and pesticide exposure [56], curcumin can preserve intestinal barrier integrity by enhancing the activity of antioxidant enzymes (e.g., SOD, CAT, and GSH-Px), downregulating the expression of pro-inflammatory cytokines (e.g., IL-1β and TNF-α), and upregulating the expression of anti-inflammatory cytokines (e.g., IL-10) as well as genes encoding intestinal tight junction proteins (e.g., Zonula occludens-1 (ZO-1), Occludin, and Claudin-1). These regulatory effects collectively contribute to improved growth performance and survival rates in broilers. A meta-analysis conducted by Hernández-Garcí et al. [70] shows that under conventional conditions, dietary supplementation with 100–200 mg/kg curcumin exerts significant beneficial effects on broiler growth performance, antioxidant capacity, intestinal morphology, and meat quality. For nano-curcumin, the recommended supplementation range is 100–400 mg/kg, with 300 mg/kg being the optimal dosage for maximizing improvements in growth performance, antioxidant capacity, and intestinal health according to Abdel-Moneim et al. [59]. In response to specific stressors or disease challenges (e.g., heat stress, coccidiosis infection), the supplementation level can be moderately increased, though an upper limit of 400 mg/kg is advised to avoid adverse effects [55,57,59,66,69]. Notably, high-dose supplementation (1000 mg/kg and above) has been shown to exert inhibitory effects on broiler growth performance suggested by Xie et al. [71]. However, according to Gharibet al. [47], under heat stress, supplementation at 1000 mg/kg has been reported to alleviate heat stress damage, enhance immunity, and improve antioxidant capacity in broilers [55].

Collectively, curcumin exerts comprehensive, multi-pathway beneficial effects in broiler production, making it a promising natural feed additive. As summarized in Table 1, dietary curcumin supplementation improves growth performance and feed efficiency [49,52], enhances intestinal health by optimizing gut microbiota and barrier function [54,55], boosts systemic antioxidant capacity [62,68], modulates immune function [57,72], mitigates stress and disease challenges [55,56,57,69], improves meat quality [58,70], and regulates lipid metabolism [61,71]. Nevertheless, some inconsistent results regarding its effective dosage have been reported in the literature [55,71], which may be associated with differences in experimental conditions and curcumin preparations. These synergistic effects are particularly pronounced under stress and disease conditions, highlighting its great application potential in broiler farming.

### 4.2. Application Research in Laying Hen Production

The application of curcumin in laying hen production has been extensively investigated [78,79,80,81,82,83]. Studies have demonstrated that dietary supplementation with an appropriate dose of curcumin can significantly improve the laying rate and egg weight of laying hens while reducing the feed–egg ratio, especially under adverse environments such as heat stress [79]. Additionally, curcumin can enhance key egg quality parameters, including eggshell thickness, eggshell strength, albumen height, and yolk color, thereby increasing the overall commercial value of eggs [78,79,80,81,83,84,85]. It can also improve the yolk color score and indirectly optimize yolk composition by regulating liver lipid metabolism-related genes [e.g., downregulating FASN and sterol regulatory element-binding protein-1(SREBP-1)] [78,80].

Intestinal health is critical for the production performance and overall well-being of laying hens. Curcumin exerts beneficial effects on intestinal health by enhancing intestinal barrier function, modulating the gut microbial community, and increasing digestive enzyme activity [82,85]. For instance, Xu et al. [82] showed that dietary curcumin supplementation can significantly improve intestinal morphology [e.g., increasing the villus height/crypt depth ratio (V/C)], promote the gene expression of digestive enzymes, and upregulate the expression of tight junction proteins (e.g., ZO-1, Claudin-1, and Occludin). Furthermore, Xu et al. [82] and Da Rosa et al. [85] showed that curcumin can increase the relative abundance of beneficial gut bacteria (e.g., *Bacteroidetes* and *Bifidobacterium*) and reduce the levels of potential pathogenic bacteria (e.g., *Escherichia coli*), thereby maintaining intestinal microecological balance. At the same time, curcumin can elevate the level of immunoglobulin in the intestines and serum, enhance the intestinal immune barrier, and thus promote nutrient absorption and overall health of hens as reported by Xu et al. [82] and Gu et al. [86].

In terms of lipid metabolism regulation, curcumin is reported to reduce the content of triglycerides and cholesterol in the liver and plasma of laying hens, reduce liver fat deposition, and inhibit fat synthesis by downregulating the expression of fatty acid synthesis-related genes [e.g., SREBP-1, FASN, and Acetyl-CoA carboxylase (ACC)] [78,80,87,88]. These effects facilitate the maintenance of lipid metabolic balance and reduce the risk of metabolic disorders such as fatty liver disease.

Oxidative stress is one of the key factors impairing the health and production performance of poultry. Particularly under high-temperature environments or chemical toxin exposure, excessive accumulation of ROS in the body can induce lipid peroxidation of cell membranes, protein denaturation, and DNA damage, thereby triggering inflammatory responses and immunosuppression [6,89]. As a potent antioxidant, curcumin is reported to significantly increase the activity of antioxidant enzymes such as SOD, GSH-Px and total antioxidant capacity (T-AOC) in laying hens, and reduce the level of oxidative damage products such as MDA [79,85,90]. This antioxidant activity is closely associated with the phenolic hydroxyl and β-diketone structures in curcumin molecules, which enable ROS neutralization through electron transfer and hydrogen atom transfer mechanisms [56]. The enhanced antioxidant capacity helps laying hens resist oxidative damage induced by heat stress, cold stress, and environmental toxins, thereby preserving bodily homeostasis.

Regarding immune regulation, numerous studies have demonstrated that curcumin can downregulate the expression of pro-inflammatory factors like pro-inflammatory cytokines (e.g., IL-1β, IL-6, and TNF-α) in the liver by inhibiting inflammatory signaling pathways such as toll-like receptor 4 (TLR4)/NF-κB, thereby alleviating heat stress-induced hepatic inflammation and DNA damage in laying hens [91]. Research has also shown that curcumin can reduce serum corticosterone concentration and heterophil/lymphocyte ratio (H/L ratio), target and ameliorate T-cell dysfunction by regulating the expression profile of serum exosomal miRNAs, decrease liver enzyme activities [e.g., alanine transaminase (ALT)], improve white blood cell counts, and restore immune homeostasis in laying hens under heat stress [92,93]. Collectively, findings from existing studies indicate that curcumin significantly promotes the health and production efficiency of laying hens through multiple synergistic mechanisms, including improving production performance and egg quality, regulating hepatic lipid metabolism, enhancing antioxidant capacity, and optimizing immune function and intestinal health (Table 2).

### 4.3. Application Research in Duck Production

As an important part of animal husbandry, the duck industry has significant economic value. However, in actual production, mycotoxins [such as aflatoxin B1 (AFB1) [94,95], ochratoxin A (OTA)] [96,97], endotoxins (such as LPS) [98,99], and heavy metals [such as arsenic trioxide (ATO)] [100,101,102] and other exogenous and endogenous harmful factors often threaten duck health, leading to reduced growth performance, immunosuppression, and multi-organ damage. With excellent antioxidant and anti-inflammatory activities, curcumin exhibits great potential in regulating duck growth, protecting intestinal function, alleviating stress damage, improving meat quality, modulating lipid metabolism and enhancing immunity [94,95,96,97,98,99,100,101,102,103,104,105].

Studies by Jin et al. [50] and Wan et al. [105] have shown that dietary supplementation with 300–500 mg/kg curcumin can significantly increase the final body weight and average daily gain (ADG), while reducing the feed-to-weight ratio (F/G) of ducks. This beneficial effect is primarily attributed to improved nutrient digestion and absorption, as well as the mitigation of growth inhibition resulting from toxins and environmental stressors [94,95,96,97,102,104,105]. In terms of meat quality, Jin et al. [50] showed that curcumin can effectively improve the T-AOC and the activities of SOD and GSH-Px, and reduce the content of MDA in duck meat, thereby inhibiting lipid and protein oxidation, and improving meat color (a* value), water-holding capacity and tenderness; at the same time, curcumin can improve the ultrastructure of muscle tissue and reduce myofiber damage by regulating mitochondrial function and energy metabolism, providing a structural basis for improving meat quality.

Curcumin has a good protective effect on organ damage caused by various stress factors. For example, LPS, a component of Gram-negative bacterial cell walls that induces acute inflammation [106], can be counteracted by curcumin through the simultaneous activation of the Nrf2-ARE antioxidant pathway and inhibition of the NF-κB pro-inflammatory pathway, thereby alleviating LPS-induced acute lung injury in ducks according to Liu et al. [99]. Additionally, Yang et al. [98] showed that curcumin mitigates LPS-induced intestinal morphological damage and barrier dysfunction by inhibiting the TLR4/NF-κB pathway.

AFB1 is recognized as one of the most toxic mycotoxins [107]. Curcumin can enhance the antioxidant capacity of the liver by activating the Nrf2/ARE pathway and inhibit the NF-κB/NLR family pyrin domain containing 3 (NLRP3) pathways to alleviate the inflammation and pyroptosis induced by AFB1 [94,103,108]. A recent mechanistic study by Su et al. [109] has revealed that curcumin can also inhibit ferroptosis through upregulation of glutathione peroxidase 4 (GPX4) and alleviate endoplasmic reticulum stress (ERS) to ameliorate hepatic lipid metabolism disorders caused by AFB1. In the intestine, Jin et al. [3], Pan et al. [95], and Jiang et al. [110] showed that curcumin can upregulate the expression of tight junction proteins (e.g., ZO-1, Occludin) and mucins (e.g., MUC2), and alleviate inflammation and pyroptosis by inhibiting the NF-κB/NLRP3 pathway, thereby repairing intestinal barrier function and regulating flora balance in AFB1-exposed ducks. Regarding renal health, Liu et al. [111] showed that curcumin can alleviate AFB1-induced nephrotoxicity in ducks by inhibiting mitochondria-mediated oxidative stress, ferritinophagy, and ferroptosis. In the spleen, curcumin can activate the Nrf2 signaling pathway, upregulate the expression of related antioxidant enzymes, and inhibit the NF-κB signaling pathway, ultimately reducing AFB1-induced inflammation in the spleen of ducklings, as demonstrated by Wan et al. [105].

OTA is a fungal toxin widely distributed in food and feed, and its multi-organ toxic effects are a major focus of toxicological research [112,113]. A previous study by Ruan et al. [97] demonstrated that dietary supplementation with 400 mg/kg curcumin can enhance the mRNA and protein expression of tight junction proteins (i.e., Occludin and tight junction protein 1 (TJP1)) while downregulating the expression of Rho-associated protein kinase 1 (ROCK1), thereby restoring intestinal barrier integrity, alleviating intestinal villus atrophy and epithelial shedding, and suppressing the release of pro-inflammatory cytokines including IL-1β and TNF-α. Furthermore, curcumin can modulate the composition of the intestinal microbiota, restore OTA-induced reductions in the abundance of butyrate-producing bacteria (e.g., *Blautia*, *Butyricicoccus*, and *Butyricimonas*), improve the balance of intestinal microbial metabolism, and thereby further sustain intestinal homeostasis according to Zhai et al. [96].

ATO, a prevalent environmental contaminant, exhibits prominent multi-organ toxicity [100,101,102]. Accumulating evidence indicates that curcumin mitigates ATO-induced nephrotoxicity [100], skeletal muscle damage [102], spleen injury [114], and neurotoxicity [101,115] by regulating the PTEN-induced kinase 1 (PINK1)/Parkin pathway and activating the Nrf2 signaling pathway. These mechanisms subsequently inhibit excessive autophagy and apoptosis, as well as ameliorate oxidative stress and metabolic disorders.

Overall, current research highlights the substantial application potential of curcumin in duck production. As demonstrated in Table 3, curcumin not only effectively improves production performance and meat quality but also broadly exerts protective effects against the toxicity induced by various common hazardous factors, including AFB1, OTA, ATO, and LPS, through multi-pathway synergistic actions.

### 4.4. Application Research in Quail Production

Quails are characterized by a short growth cycle, high reproductive efficiency, and their meat and egg products exhibit high nutritional value [116]. However, in intensive breeding processes, quails often face challenges such as heat stress [117], cold stress [118], and disease infection [119], which affect their production performance and product quality. As a natural plant-derived extract, curcumin has garnered increasing attention for its application potential in quail production, owing to its diverse biological activities such as antioxidant, anti-inflammatory, immunomodulatory, and growth-promoting properties [120,121,122]. Curcumin exerts comprehensive beneficial effects on quail production performance, stress resistance, intestinal health, lipid metabolism and immune function, and the specific regulatory effects and mechanisms are summarized in Table 4.

In terms of improving production performance, Liu et al. [120] reported that dietary supplementation with 200 mg/kg curcumin in late-laying quails can reduce mortality, increase eggshell thickness and strength, decrease crude fat content in eggs, and elevate the proportions of crude protein and ash. Under cold stress conditions, Marchiori et al. [123] showed that supplementation with 30 mg/kg free curcumin or 10 mg/kg nanoencapsulated curcumin can improve laying rate, optimize feed conversion rate (FCR), enhance yolk brightness and yellow intensity, and reduce lipid peroxidation level of quails. Saraswati [124] demonstrated that curcumin supplementation in combination with the hepatitis B vaccine (12 mg/bird/day) can increase egg weight, albumen content and Haugh unit, while reducing cholesterol and fat content in quail eggs [124].

Curcumin can improve the stress resistance and antioxidant capacity of quails. For instance, Reda et al. [121] showed that a diet supplemented with nano-curcumin (0.2 g/kg) can significantly increase SOD and glutathione (GSH) activities, reduce MDA levels, as well as increase serum immunoglobulin (IgG, IgM) levels and complement activity, thereby improving the disease resistance of quails. Sahin et al. [122] showed that dietary curcumin at 200–400 mg/kg under heat stress conditions can reduce MDA content in serum, muscle and liver, increase the activity of antioxidant enzymes such as SOD and CAT, inhibit the expression of NF-κB and heat shock protein 70 (HSP70), and alleviate oxidative damage by regulating the Nrf2/HO-1 pathway [122].

Curcumin can modulate the intestinal flora structure of quails. For instance, Liu et al. [120] and Reda et al. [121] showed that a diet supplemented with curcumin can increase the Shannon diversity index, regulate the abundance of phyla such as Actinobacteria and Firmicutes, promote the proliferation of beneficial bacteria (e.g., *Lactobacillus*), and suppress pathogenic bacteria (e.g., *Salmonella*).

In addition, has a certain regulatory effect on lipid metabolism in quails [120,121]. For instance, Liu et al. [112] demonstrated that curcumin can reduce liver fat accumulation by regulating the expression of acyl-CoA oxidase 2 (ACOX2) and stearoyl-CoA desaturase 1 (SCD1) proteins, and reduce serum triglyceride (TG), total cholesterol (TC) and low-density lipoprotein (LDL) levels in quails; Reda et al. [121] demonstrated that nano-curcumin can further increase high-density lipoprotein (HDL) levels and optimize lipid metabolism in quails, and its regulation of lipid metabolism is closely associated with the improvement of quail body health and product quality.

## 5. Limiting Factors and Future Research Directions of Curcumin Application

### 5.1. Application Limiting Factors

Despite the considerable potential of curcumin in poultry production, its large-scale implementation is hindered by multiple practical limitations, as widely documented in recent studies, primarily stemming from mismatches between its intrinsic properties and field application scenarios.

(1) Low bioavailability is a core scientific problem restricting the efficacy of curcumin [6]. According to previous investigations, due to its hydrophobic structure, curcumin exhibits poor solubility in the aqueous intestinal environment, is susceptible to degradation by intestinal microbiota, and undergoes rapid hepatic biotransformation and elimination, resulting in significantly lower bioavailability in animals compared to conventional antibiotic additives [7,16]. It has been documented that although novel formulations (e.g., nanoemulsions, cyclodextrin inclusions, and metal complexes) can moderately enhance solubility and stability [8,9,10,125], targeted delivery and sustained release remain challenging. Consequently, high dosages are required to achieve desired biological effects, increasing application costs and potentially inducing physiological stress in poultry.

(2) Insufficient chemical stability poses a critical technical barrier to industrial application. As reported by previous researchers, the β-diketone moiety in curcumin is highly sensitive to light, heat, and alkaline conditions, leading to isomerization and degradation during high-temperature feed pelleting and long-term storage [7,8,13,14,15]. This compromises active ingredient retention and efficacy.

(3) Complex dose–effect relationships and inadequate safety evaluations impede precise application. According to available reports, optimal curcumin dosages exhibit marked species specificity, breed differences, and dependence on rearing conditions. Optimal ranges for different poultry species (e.g., broilers, layers, ducks, quails) remain unelucidated, with a paucity of systematic data supporting dose adjustments under stress or disease conditions [55,59,70,82]. It has been reported that high-dose curcumin (≥1000 mg/kg) may inhibit poultry growth [71]. Long-term supplementation effects on reproductive performance, metabolic organ function, and offspring health are poorly characterized, restricting precise application and risk management across breeding scenarios.

(4) Immature industrialization and cost challenges hinder market penetration. Curcumin extraction is complex, with high costs for high-purity raw materials. Large-scale production of advanced formulations (e.g., nanoencapsulation, metal complexation) remains suboptimal, characterized by low efficiency and high costs [8,9,10]. Moreover, the lack of standardized preparation and quality control standards also contributes to inconsistent outcomes in practical applications, which is an important gap for future industrial development. This renders curcumin-containing feeds significantly more expensive than conventional alternatives, lacking market competitiveness and limiting adoption by small-to-medium breeding enterprises.

### 5.2. Future Research Directions

To overcome the above limitations, primarily stemming from curcumin’s intrinsic physicochemical properties, future research should focus on formulation innovation, mechanism clarification, standard establishment and industrial optimization, so as to promote the transformation of curcumin from laboratory research to large-scale industrial application.

First, innovation in preparation technology will improve inherent bioavailability defects. To enhance market acceptance, develop intestinal-specific responsive coating preparations according to poultry intestinal characteristics to achieve targeted and sustained release; explore the synergistic application of curcumin with plant essential oils, probiotics, etc., to reduce the dosage of single components; and develop low-cost carriers derived from agricultural wastes to lower production costs, thereby improving economic viability.

Second, deepen mechanistic research to support precise applications. Study the action mechanisms of curcumin in different poultry breeds, physiological stages and stress states, focusing on the regulatory pathways of intestinal microbial metabolites and differential actions in stress signaling pathways. Clarifying the key targets to improve production performance will provide a solid theoretical basis for its targeted application in the market.

Third, establish a standardized application system to ensure quality and stability. Conduct multi-center and large-sample field experiments to determine the optimal dosage, application cycles and withdrawal periods of curcumin for different poultry under different conditions. Formulate strict quality control standards for curcumin feed additives and build a full-chain traceability system. These measures are essential for ensuring product consistency and gaining regulatory recognition and market trust.

Fourth, optimize the industrialization process to reduce costs and enhance competitiveness. Improve the yield and purity of curcumin by optimizing green extraction technologies such as ultrasonic-assisted extraction and microwave extraction; promote continuous and automated large-scale preparation processes to reduce energy consumption; and carry out comprehensive economic and environmental benefit evaluations to provide data support for market promotion.

In addition, strengthen safety and environmental impact assessment. Systematically study the effects of long-term high-dose addition on poultry health and the ecological environment, and ensure food safety and ecological security during the large-scale application of curcumin.

## 6. Conclusions

Curcumin is a promising antibiotic alternative for poultry production, exerting antioxidant, anti-inflammatory and metabolic regulatory effects via the Nrf2/ARE and NF-κB pathways. It improves production performance, product quality and stress/disease resistance in broilers, laying hens, ducks and quails, with nano-formulations boosting its efficacy. Limited by low bioavailability, poor stability, non-standardized dosages and high industrial costs, future research should focus on preparation innovation, mechanism clarification, standardized application and industrial process optimization. As a green feed additive, curcumin has broad prospects for poultry industry sustainability under antibiotic-free and zinc restriction policies.

## Figures and Tables

**Table 1:** Overview of the main biological effects of curcumin in broiler chickens.

Main Effect	Specific Outcomes/Mechanisms	References
Improved Growth Performance	Increased daily weight gain, better FCR, higher carcass yield	[49,51,52,54,55,56,57,59,69,70,72,73,74,75,76]
Enhanced Intestinal Health	Increased villus height, reduced crypt depth, improved intestinal barrier, modulated gut microbiota	[54,55,57,59,60,69,70,73,76]
Antioxidant Activity	Increased SOD, CAT, GSH-Px, T-AOC; reduced MDA; improved oxidative stability in tissues	[49,51,52,53,55,56,57,59,62,65,66,67,68,69,70,72,76]
Immune Modulation	Elevated immunoglobulins (IgG, IgM, IgA), improved immune organ development, reduced inflammatory cytokines	[49,51,54,56,57,59,72]
Stress/Disease Resistance	Mitigated effects of heat stress, coccidiosis, pesticide and mycotoxin exposure	[51,55,56,57,59,62,63,64,65,66,67,68,69]
Improved Meat Quality	Enhanced meat color (L*, a*, b*), water-holding capacity, tenderness, reduced fat and MDA, better amino acid, fatty acid, and volatile compound profiles	[58,60,61,64,65,70,77]
Lipid Metabolism Regulation	Lowered serum cholesterol, LDL, triglycerides, reduced abdominal fat, downregulated lipogenesis genes	[59,60,71]

a*: redness; b*: yellowness; CAT: Catalase; FCR: feed conversion ratio; GSH-Px: glutathione peroxidase; IgA: immunoglobulin A; IgG: immunoglobulin G; IgM: immunoglobulin M; L*: lightness; LDL: low-density lipoprotein; MDA: Malondialdehyde; SOD: superoxide dismutase; T-AOC: total antioxidant capacity.

**Table 2:** Overview of the main biological effects of curcumin in laying hens.

Main Effect	Specific Outcomes/Mechanisms	References
Growth Performance/Egg Quality	Improve egg production rate, egg weight, eggshell strength, and egg quality; improve the egg yolk color score	[78,79,80,81,83,84,85]
Intestinal health	Improve intestinal structure, enhance barrier function, optimize gut microbiota, and strengthen absorption and immunity	[82,85,86]
Antioxidant activity	Increase the activity of enzymes such as SOD and GSH-Px, and reduce MDA	[79,85,90]
Immune modulation	Enhance immunoglobulins, inhibit inflammatory factors, and regulate immune signaling pathways	[79,82,83,91,92,93]
Lipid Metabolism Regulation	Lower TG/cholesterol, inhibit the expression of genes involved in fat synthesis, and reduce fat deposition	[78,80,84,87,88]

GSH-Px: glutathione peroxidase; MDA: Malondialdehyde; SOD: superoxide dismutase; TG: triglycerides.

**Table 3:** Overview of the main biological effects of curcumin in ducks.

Main Effect	Specific Outcomes/Mechanisms	References
Improved Production Performance	Increased final BW, WG, and FI	[50]
	Prevented the decrease in BW and ADG induced by OTA	[97]
	Alleviated growth retardation induced by AFB1	[95,105,110,111]
	Attenuated ATO-induced body weight loss	[100,101,102,114,115]
Enhanced Intestinal Health	Improved intestinal morphology (VH↑, CD↓, V/C↑), decreased permeability serological index (DAO and D-LA)	[95,98,110]
	Strengthened intestinal barrier (ZO-1, Occludin, Claudin-1)	[95,97,98]
	Regulated gut microbiota (increased diversity, beneficial bacteria)	[96,110]
	Increased mucin secretion (MUC2) and goblet cell count	[95]
Antioxidant Effects	Activated Nrf2-ARE signaling pathway, increased antioxidant enzyme activities (SOD, CAT, GSH-Px, HO-1), decreased oxidative stress markers (MDA, H_2_O_2_)	[3,50,94,97,98,99,100,101,102,105,110,111]
Immune regulation	Modulated TLR4/NF-κB signaling pathway, inhibited NLRP3 inflammasome activation and pyroptosis, reduced pro-inflammatory cytokines (TNF-α, IL-1β, IL-6, IFN-γ), increased serum immunoglobulins (IgA, IgG, IgM)	[3,95,98,99,101,103,105,108,110,114,115]
Improved Meat Quality	Improved meat color (increased a*), enhanced water-holding capacity (reduced drip/cooking loss), inhibited lipid and protein oxidation (reduced TBARS, carbonyls), improved tenderness (reduced shear force)	[50]
Regulation of Lipid Metabolism	Activated LKB1-AMPK signaling pathway, reduced liver triglyceride (TG) and total cholesterol (T-CHO), inhibited SREBP1c expression, alleviating hepatic steatosis	[96,109,110]
Alleviation of Endoplasmic Reticulum Stress	Reduced expression of ER stress markers (GRP78, CHOP), modulated UPR signaling (PERK, IRE1α, ATF6)	[109]
Anti-stress Effects	Mitigated oxidative, inflammatory, and cellular damage from various stressors (mycotoxins, heavy metals, LPS)	[94,95,96,97,98,99,100,101,102,105,108,109,110,111,114,115]

a*: redness; ADG: Average daily gain; AFB1: aflatoxin B1; AMPK: adenosine monophosphate-activated protein kinase; ATO: arsenic trioxide; ARE: antioxidant response element; BW: body weight; CAT: catalase; CD: crypt depth; CHOP: C/EBP homologous protein; Claudin-1: Claudin-1; DAO: Diamine Oxidase; D-LA: D-Lactic Acid; FI: feed intake; GSH-Px: glutathione peroxidase; GRP78: Glucose-Regulated Protein 78; HO-1: Heme Oxygenase-1; H_2_O_2_: Hydrogen Peroxide; IFN-γ: Interferon-γ; IL-1β: interleukin-1β; IL-6: interleukin-6; IRE1α: Inositol-Requiring Enzyme 1α; LPS: lipopolysaccharide; LKB1: Liver Kinase B1; MUC2: mucin 2; MDA: Malondialdehyde; NF-κB: nuclear factor-κB; NLRP3: NLR Family Pyrin Domain Containing 3; Nrf2: nuclear factor erythroid 2-related factor 2; Occludin: Occludin; OTA: Ochratoxin A; PERK: Protein Kinase R-Like Endoplasmic Reticulum Kinase; SOD: superoxide dismutase; SREBP1c: sterol regulatory element-binding protein 1c; TBARS: Thiobarbituric Acid Reactive Substances; TNF-α: tumor necrosis factor-α; T-CHO: total cholesterol; TG: triglycerides; TLR4: toll-like receptor 4; UPR: Unfolded Protein Response; VH: villus height; V/C: villus height/crypt depth ratio; ZO-1: zonula occludens-1.

**Table 4:** Summary of curcumin’s main effects in quails.

Main Effect	Specific Outcomes/Mechanisms	References
Growth Performance/Egg Quality	Reduced mortality; decreased egg fat/cholesterol; increased eggshell strength/thickness, egg weight, albumen content, Haugh unit and yolk color; elevated egg crude protein/ash; improved FCR	[120,121,122,123,124]
Intestinal health	Increased intestinal flora Shannon index; regulated Actinobacteria/Firmicutes abundance; promoted *Lactobacillus*, suppressed *Salmonella*	[120,121]
Antioxidant Capacity	Reduced serum, muscle and liver MDA; increased SOD, CAT and GSH-Px activities; inhibited NF-κB and HSP70	[120,121,122,123]
Immune modulation	Increased serum IgG/IgM and complement activity	[121]
Lipid Metabolism Regulation	Reduced liver fat deposition; regulated ACOX2, SCD1; decreased serum TG, TC, LDL; nano-curcumin elevated serum HDL	[120,121]
Stress/Disease Resistance	Mitigated effects of heat stress and cold stress; improved overall disease resistance in quails	[121,122,123]

ACOX2: Acyl-CoA oxidase 2; CAT: catalase; FCR: feed conversion rate; GSH-Px: glutathione peroxidase; HDL: high-density lipoprotein; HSP70: heat shock protein 70; IgG: immunoglobulin G; IgM: immunoglobulin M; LDL: low-density lipoprotein; MDA: Malondialdehyde; NF-κB: nuclear factor-κB; SCD1: stearoyl-CoA desaturase 1; SOD: superoxide dismutase; TC: total cholesterol; TG: triglycerides.

## Data Availability

No new data were created or analyzed in this study. Data sharing is not applicable to this article.

## Abbreviations

The following abbreviations are used in this manuscript:

ACC	Acetyl-CoA carboxylase
ACOX2	Acyl-CoA oxidase 2
AD	Alzheimer’s disease
ADG	Average daily gain
AFB1	Aflatoxin B1
ALT	Alanine transaminase
ARE	Antioxidant response element
ATO	Arsenic trioxide
CAT	Catalase
CD	Crypt depth
DAMPs	Damage-associated molecular patterns
ERS	Endoplasmic reticulum stress
ERK	Extracellular regulated protein kinases
FASN	Fatty acid synthase
FCR	Feed conversion ratio
F/G	Feed-to-weight ratio
GSH	Glutathione
GSH-Px	Glutathione peroxidase
GPX4	Glutathione peroxidase 4
HDL	High-density lipoprotein
H/L ratio	Heterophil/lymphocyte ratio
HO-1	Heme oxygenase
HSP70	Heat shock protein 70
IκBα	Nuclear factor of kappa light polypeptide gene enhancer in B-cells inhibitor α
IgG	Immunoglobulin G
IgM	Immunoglobulin M
IKK	IκB kinase
IL-1β	Interleukin 1β
IL-6	Interleukin 6
JNK	c-Jun N-terminal kinase
Keap1	Kelch-like ECH-associated protein 1
LDL	Low-density lipoprotein
LPS	Lipopolysaccharide
MAP3Ks	Mitogen-activated protein kinase kinase kinases
MAPK	Mitogen-activated protein kinase
MDA	Malondialdehyde
MGAM	Maltase-glucoamylase
MUC2	Mucin 2
NF-κB	Nuclear factor-κB
NLRP3	NLR family pyrin domain containing 3
NQO1	NADP(H): quinone oxidoreductase
Nrf2	Nuclear factor erythroid 2-related factor 2
OTA	Ochratoxin A
PAMPs	Pathogen-associated molecular patterns
PPAR-γ	Peroxisome proliferator-activated receptor γ
PINK1	PTEN-induced kinase 1
ROS	Reactive oxygen species
ROCK1	Rho-associated protein kinase 1
SCD1	Stearoyl-CoA desaturase 1
SI	Sucrase-isomaltase
SOD	Superoxide dismutase
SREBP-1	Sterol regulatory element-binding protein-1
TAK1	Transforming growth factor-activated kinase
TC	Total cholesterol
TG	Triglycerides
T-AOC	Total antioxidant capacity
TLR4	Toll-like receptor 4
TNF-α	Tumor necrosis factor-α
TJP1	Tight junction protein 1
UAE	Ultrasonic-assisted extraction
VH	Villus height
ZO-1	Zonula occludens-1
